# Examination of Provider and Patient Knowledge, Beliefs, and Preferences in Integrative Oncology at a National Cancer Institute-Designated Comprehensive Cancer Center

**DOI:** 10.1089/imr.2021.0004

**Published:** 2022-06-24

**Authors:** Jennifer S. Mascaro, Almira Catic, Meha Srivastava, Maggie Diller, Shaheen Rana, Cam Escoffery, Viraj Master

**Affiliations:** ^1^ Department of Family and Preventive Medicine, Emory University School of Medicine, Atlanta, GA, USA.; ^2^ Winship Cancer Institute, Emory University, Atlanta, GA, USA.; ^3^ Department of Surgery, Emory University School of Medicine, Atlanta, GA, USA.; ^4^ Department of Behavioral, Social, Health Education Sciences, Rollins School of Public Health, Emory University, Atlanta, GA, USA.; ^5^ Department of Urology, Emory University, Atlanta, GA, USA.

**Keywords:** integrative oncology, survivorship, implementation science, barriers, facilitators, comprehensive cancer center

## Abstract

**Purpose:**

The use of integrative approaches for symptom management is highly prevalent among patients undergoing cancer treatment and among cancer survivors and is increasingly endorsed by clinical practice guidelines. However, access to and implementation of integrative oncology (IO) approaches are hindered by barriers at multiple levels, including logistic, geographic, financial, organizational, and cultural barriers. The goal of this mixed-method study was to examine oncology provider and patient knowledge, beliefs, and preferences in IO to identify facilitators, barriers, and recommendations for implementation of IO modalities.

**Materials and Methods:**

Data sources included patient surveys and provider semistructured interviews. Patients were in active treatment (*n* = 100) and survivors (*n* = 100) of heterogeneous cancer types. Patient and survivor surveys interrogated: (1) interest in types of IO approaches; and (2) preferences for delivery modality, frequency, and location. Providers (*n* = 18) were oncologists and nurse navigators working with diverse cancer types. Interviews queried their knowledge of and attitudes about IO, about their patients' needs for symptom management, and for recommendations for implementation of IO approaches in their clinic. We used the Consolidated Framework for Implementation Research framework to systematically analyze provider interviews.

**Results:**

The primary interests reported among actively treated patients and survivors were massage therapy, acupuncture, and wellness/exercise. Most patients expressed interest in both group and individual sessions and in telehealth or virtual reality options. Emergent themes from provider interviews identified barriers and facilitators to implementing IO approaches in both the internal and external settings, as well as for the implementation process.

**Conclusion:**

The emphasis on mind–body interventions as *integrative* rather than *alternative* highlights the importance of interventions as evidence-based, comprehensive, and integrated into health care. Gaining simultaneous perspectives from both patients and physicians generated insights for the implementation of IO care into complex clinical systems within a comprehensive cancer center.

## Introduction

In 2019, more than 16.9 million Americans were cancer survivors, and that number is projected to grow to more than 20 million in 2026.^1^ Epidemiological studies find that survivors of almost all types of cancer have significantly poorer mental health^2^ and health-related quality of life (QOL)^3^ than people without cancer. Both depression and QOL during and after cancer treatment predict the mortality rate, independent of, and sometimes better than,^4^ clinical variables of disease.^5,6^ Effective evidence-based interventions to improve psychosocial well-being and long-term QOL for cancer survivors are imperative to advancing cancer care.^7,8^

Integrative oncology (IO) refers to the field that uses evidence-informed and patient-centered interventions alongside conventional treatment modalities as part of comprehensive cancer care. Integrative approaches are used before, during, and beyond cancer treatment with the aim of improving clinical outcomes, promoting QOL, and optimizing health and well-being.^9^ The use of integrative approaches to improve well-being and QOL is prevalent and increasing among cancer patients, with estimates of 49% to 91% of cancer survivors using integrative approaches at some point.^10–12^

Given the importance of identifying and evaluating interventions to improve well-being and QOL among patients and survivors and considering the prominence and promise of IO in comprehensive cancer care, it is imperative to identify the barriers and facilitators to integrating IO approaches into treatment and beyond. Commonly cited barriers to IO identified in previous research include patients' lack of time, lack of awareness of the benefits of IO, physical and psychosocial symptoms, lack of interest or motivation, and cost.^13–16^ In some complex health systems, the barriers to IO can prove more challenging than is building the evidence base to justify the value of IO approaches.^16^

Toward this goal, examining both patient and provider perspectives is critical to identify potential mismatches in interest, beliefs, or preferences.^17^ For example, a recent report found that, of the patients who report using complementary and integrative therapies, only 57% discussed the topic with their oncologist or another provider.^12^ Another study found that although the most common way patients undergoing active treatment learn about IO approaches is from their oncologist, more than half of the patients reported that their physician did not offer integrative options to them and only 15% reported talking to their physician about integrative approaches.^18^ The goal of this mixed-method study was to evaluate oncology provider and patient knowledge, beliefs, and preferences in IO to identify facilitators, barriers, and recommendations for implementation of IO modalities.

## Materials and Methods

### Overview

This mixed-method study was conducted as formative research at the commencement of an Integrative Oncology and Survivorship program in a National Cancer Institute (NCI)-designated Comprehensive Cancer Center in the Southeast United States. Surveys and interviews were developed as a comprehensive program needs assessment, designed to identify needs and priorities for the purpose of making decisions about the allocation of resources (financial, temporal, space, and personnel).^19^ Data sources included patient and survivor surveys collected by phone and semistructured interviews with oncology providers. Because data were anonymized and gathered for program evaluation, the institutional review board determined that institutional review and consent were not necessary. No incentives were given for participation.

### Participants

Patients were adults (18 or older) in active treatment (*n* = 100) and survivors (*n* = 100) of heterogeneous cancer types. For the active patients, we identified and contacted patients from patient lists provided by the genitourinary (GU), gastrointestinal (GI), breast, melanoma, lymphoma, and head and neck clinics. For survivors, we identified and contacted patients from the survivorship clinic (heterogenous cancer types, unspecified for this study). Some participants in the survivor group had been pediatric cancer patients, but all were 18 or older at the time of the survey. Providers (*n* = 18) were oncologists and nurse navigators working with diverse cancer types. The selection of providers was determined by disease group, with an effort to ensure that most oncologic diseases are represented. Providers were interviewed from lymphoma, myeloma, GI, melanoma, brain, lung, breast, GU, head and neck, bone marrow transplant, the ambulatory infusion center, survivorship, nursing navigation, and pediatrics.

### Patient and survivor surveys

To develop the survey, we conducted an informal market analysis to determine the services that are currently offered in the market local to the cancer center as well as by other cancer centers with similar programs. We developed questions based on website reviews, interviews, and visits to collaborating cancer centers, including Memorial Sloan Kettering, Miami Cancer Institute, MD Anderson, Atrium Health Levine Cancer Institute, Cancer Treatment Centers of America, and Cleveland Clinic. In addition, the survey included logistical questions that emerged during program development in discussion with cancer center leadership. Patient and survivor surveys were administered by phone using a convenience sampling approach.

We administered the same survey to both groups, which interrogated the following: (1) familiarity with IO using a Likert scale from 0 (“Not at all familiar”) to 10 (“Extremely familiar”), (2) interest in types of IO approaches, and (3) preferences for delivery modality, frequency, and location (see Supplementary Data for patient/survivor questions). The survey also included an option to provide an open-ended comment.

### Provider interviews

Oncology providers were contacted via email and asked to participate in a needs assessment for IO and Survivorship program planning and development. Only one provider was interviewed in person, while 17 providers were interviewed virtually via Zoom (due to the COVID-19 pandemic). Interviews queried their knowledge of and attitudes about IO, about their patients' needs for symptom management, and for recommendations for implementation of IO approaches in their clinic (see Supplementary Data for provider questions). Interviews were transcribed by the researcher who conducted the interviews (A.C.).

We used the Consolidated Framework for Implementation Research (CFIR) framework to systematically examine provider interviews.^20^ CFIR defines five theory-based domains that are associated with the effective adoption, implementation, and maintenance of interventions: (1) Intervention characteristics, (2) Outer setting, (3) Inner setting, (4) Characteristics of individuals, and (5) Process. Each domain has a set of constructs associated with it, and we used these constructs as independent codes to get a granular understanding of the factors identified in each of these five theoretical domains.

Each interview was independently coded by two researchers (coders were J.S.M., C.E., S.R.). The entire research team discussed codes to reconcile any coding differences and to ensure concordance and reliability. In addition, themes were given a strength score based on their salience in the interviews (i.e., relative frequency with which each code emerged in the interviews). Strong themes were those that arose in 70% or more of the interviews, moderate themes were those that arose in 45–69% of the interviews, and weak themes arose in less than 45% of the interviews.

## Results

### Survivor and active patient survey

**FIG. 1.**
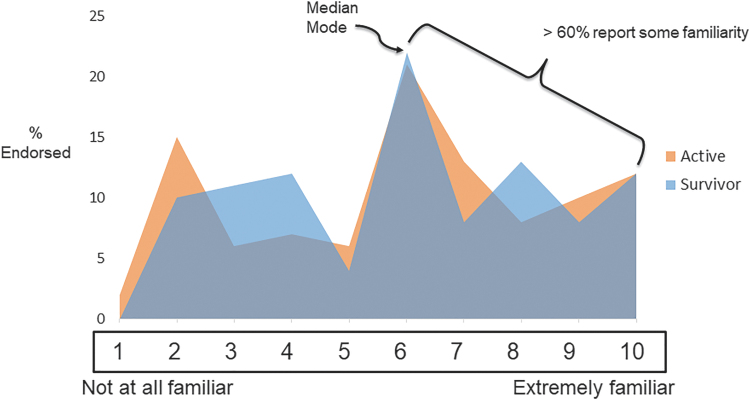
Patient-reported familiarity with IO approaches. The median and modal level of familiarity with IO for both survivors and active patients was 5 (range = 0–10), with 63% and 64% of survivors and active patients reporting familiarity (a score of 5 or higher). IO, integrative oncology.

**FIG. 2.**
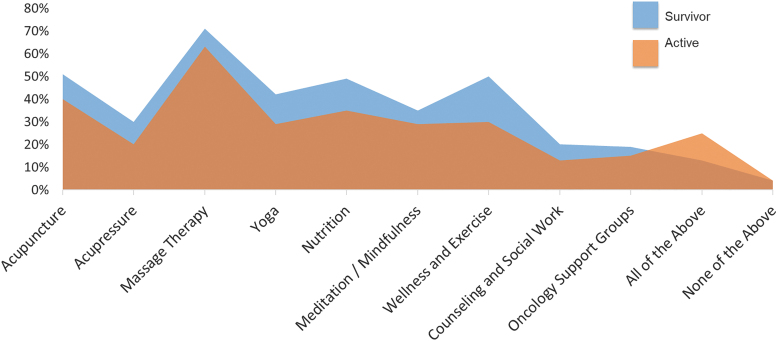
Patient-reported endorsement rates for interest in specific IO approaches.

The median and modal level of familiarity with IO for both survivors and active patients was 5 (range = 0–10), with 63% and 64% of survivors and active patients reporting familiarity (a score of 5 or higher) (Fig. 1). For both groups, the highest level of interest was in massage therapy (71% of survivors, 63% of active treatment), acupuncture (51% of survivors, 40% of active treatment), wellness/exercise (50% of survivors, 30% of active treatment), and nutrition (49% of survivors, 35% of active treatment) (Fig. 2).

Regarding preferences (Table 1), a majority of both the survivors and active patients reported that they would prefer to have both individual and group therapy options, that they would be able to participate once/week, and that the optimal session length would be 60 min. Most respondents in both groups reported that they would prefer to attend sessions closer to home rather than at the cancer institute, and that they would be interested in using technologies such as virtual reality or telehealth.

**Table 1.** Patient Preferences for Integrative Oncology Intervention Format, Frequency, Location, and Modality

	Survivor (%)	Active (%)
How often would you be able to participate in IO therapy?		
Once per week	57	47
Twice per week	21	35
Three times per week	7	5
More than three times per week	2	1
Not sure	13	12
How much time would you be able to dedicate to a single therapy session?		
Less than 30 min	3	2
30 min	19	13
45 min	24	24
60 min	45	47
More than 60 min	5	5
Not sure	4	9
Where would you most likely attend IO therapy?		
Winship Cancer Institute	24	32
Closer to home	38	34
Online	11	3
No preference	27	31
If interested in IO therapy, when would be the best time for you to attend sessions?		
Day of treatment/appoint	7	12
Weekdays, working hours	14	11
Weekdays in the evenings	17	22
Weekends	16	11
No preference	46	44
Would you be interested in participating in IO therapy using technology (e.g., virtual reality or telehealth)?		
Yes	53	47
No	19	17
Not sure	28	36

IO, integrative oncology.

Eighteen active patients and 14 survivors provided open-ended comments at the end of the survey. The most common comment was enthusiasm for IO programming, with six active patients and four survivors expressing interest (e.g., “It's great, will do anything to help with symptoms.” “So necessary as we move towards mind body connection, awareness and importance and emphasizes that survival is possible and reinforces hope.”). Seven active patients mentioned that they already use some form of IO modality (e.g., “Already using massage once a week.”). One active patient and two survivors highlighted the importance of social support (e.g., “Groups should be formed based on similar diagnoses, specific side effects, and other similarities; I'd be much more likely to participate and gain from this if there was some shared experience between myself and group-mates beyond just having cancer.”).

Three survivors advocated for the importance of accessibility (e.g., “I have hearing issues; online is not accessible and I really want in-person options.” “It's really important to make sure this is accessible for physically disabled people.” “Please accommodate for special needs; include ASL interpreters.”). One patient stated that he or she would do whatever his or her doctor recommends. Only two people surveyed included negative comments, with one active patient saying he or she was not interested in IO programming, and one survivor expressing concern about costs (“I am concerned about prices for these therapies, otherwise all for it.”).

### Provider interviews

Emergent themes and their relative strength are reported in Table 2A–E according to the CFIR construct. With respect to the characteristics of IO interventions (Table 2A), there was a common emphasis on the importance of establishing an evidence base to get buy-in and to ensure that IO approaches were easily accessible, well-packaged, and free or inexpensive. The strongest theme was related to intervention complexity, and four providers (22%) said that it would be critical to offer in-clinic services that patients could use during downtime. With respect to outer setting (Table 2B), the most stated priority was to aim for specificity in addressing the needs of specific patient groups in terms of symptom management, pain management, and whole-person well-being.

**Table 2.** Themes Identified in Provider Interviews, Listed According to Consolidated Framework for Implementation Research Domain and Color Coded According to the Strength of the Theme: Dark Gray (White Text) = Strong; Medium Gray = Moderate, 20–59%; Pale Gray = Weak, <20%

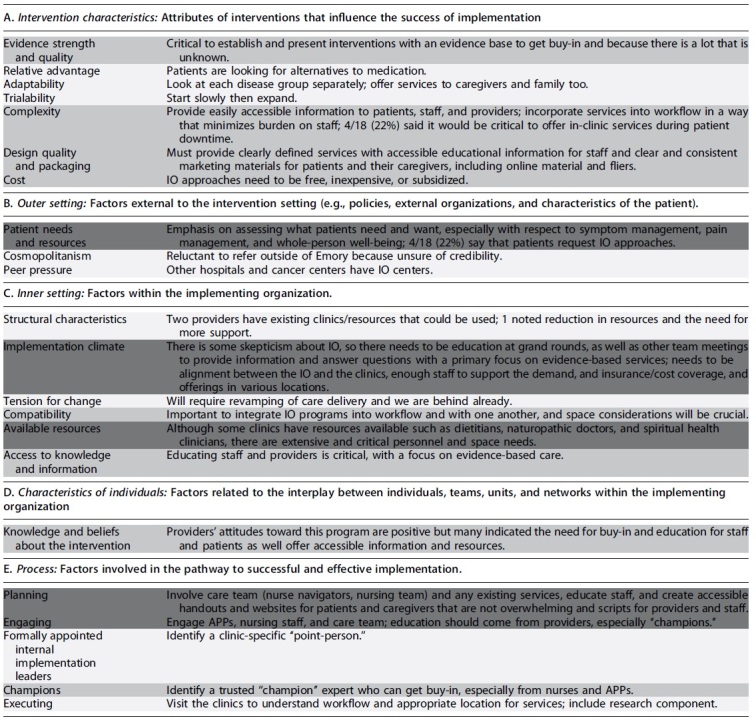

APPs, advanced practice providers.

Several providers noted that their patients requested integrative approaches to manage their symptoms. With respect to inner setting (Table 2C), the most frequently stated theme was related to education of staff and providers, especially with respect to the evidence base for IO therapies and interventions. Several providers noted the importance of utilizing existing resources and named the available resources in their clinics, such as dietitians, naturopathic doctors, and spiritual health clinicians; however, extensive and critical personnel and space shortages were also a strong theme. In terms of characteristics of individuals (Table 2D), there was a high degree of positive attitudes toward IO expressed by the providers we interviewed.

However, many stakeholders expressed a lack of knowledge and a need for education around the evidence base for IO, especially to get buy-in from other providers and staff in their clinic. With respect to process (Table 2E), involving and engaging advanced practice providers (APPs), nursing staff, and care team with education were a strong theme for the process of implementing IO. Along those lines, several providers mentioned the importance of creating handouts and websites for patients and caregivers and scripts for providers and staff.

Ultimately, providers recommended making IO easily accessible; focusing on evidence-based services; educating providers, staff, patients, and caregivers; and focusing on symptom management without overburdening the patients and providers. Providers also must have tools such as decision trees, menu of services, marketing materials, and IO experts and champions represented in clinics. They stated that the success of an IO program will rely on seamless integration in current clinic flow, the reduction of burden on patients by utilizing patients' downtime and by reducing or eliminating costs, and clearly defined offerings with staff to support the demand of patients.

## Discussion

In this large, mixed-method study of patients being actively treated, cancer survivors, and diverse oncology providers, we found broad agreement among all groups that patients have relatively high levels of familiarity and interest in IO approaches. There was also a high degree of overlap in the preferences and interests between active patients and survivors. While the provider interviews uncovered numerous recommendations for implementation, at least one area of potential mismatch between providers and patients was identified, with providers appearing to overestimate patients' desire to engage with IO approaches in the clinic compared with close to their home.

Previous research highlights the importance of examining patient perceptions and beliefs in the context of IO. The deliberate shift to the term *integrative* as opposed to *alternative* medicine emphasizes the role of these interventions as complementary to conventional treatment rather than as alternatives to or as conflicting with medical treatment.^9,21^ However, a recent survey found that 38% of Americans believe that cancer can be cured by alternative therapies alone, a belief held more often by younger people (age 18–37).^22^ Another study found that more than 85% of patients using integrative approaches reported that they used it as a cancer treatment and “to help fight the cancer.”^23^ Patients with common cancers, including nonmetastatic breast, prostate, lung, or colorectal cancer, who chose alternative medicine without any conventional cancer treatment had a mortality rate 2.5 times that of case-matched patients using conventional treatment regimens.^24^

Together, these findings highlight the importance of understanding patient preferences, as well as examining whether there are mismatches between patient and provider preferences and beliefs.

Most patients queried in both groups reported familiarity with IO approaches. In terms of interest, both active patients and survivor groups were most interested in acupuncture, massage, nutrition, and wellness/exercise. This is consistent with a previous study that found high levels of interest in massage.^18^ The majority of both groups reported that they would prefer to have both individual and group therapy options, that they would be able to participate once/week, that the optimal session length would be 60 min, and that they would prefer to attend sessions closer to home rather than at the cancer institute. The last preference is consistent with a recent study that found that the majority of patients surveyed about their preferred location for yoga would be a studio close to their home, which had higher rates of endorsement than in-clinic services.^13^

In this study, we applied CFIR in the early stages of the development of an IO program to identify factors in the inner and outer environment that could facilitate the implementation of new interventions. While CFIR is among the most commonly used and influential frameworks for implementation research,^20^ only a handful of studies in the field of IO have used rigorous theory-driven frameworks to examine the implementation of IO approaches. CFIR has been used to evaluate facilitators and barriers and the financial sustainability of group medical visits to increase access to IO modalities and patients with heterogeneous cancer types.^25^ Others have used CFIR to evaluate a stepped-care Psycho-Oncology program to examine which baseline implementation constructs predicted implementation success.^26^

Another study used the CFIR to evaluate the implementation of a group-based psychoeducational intervention for people affected by pancreatic cancer.^27^ The continued use of this framework for examining the implementation process will be critical for successful implementations.

With respect to provider beliefs about IO interventions, the strongest themes were around the importance of establishing and conveying the evidence base to educate and establish buy-in from oncologists. Most of the providers reported that IO approaches are acceptable and that their patients commonly request IO therapies. These are important findings related to high acceptability for the development of IO within our cancer center. The strongest themes in terms of the interventions themselves were that IO approaches must be clearly defined and explained to patients and that they must be cost free or inexpensive. These two themes are consistent with findings emerging from the systematic study of barriers to IO, namely, that lack of patient awareness and cost are common barriers to access.^16,28^

Previous studies have found that oncologists report that their own lack of knowledge about integrative approaches is a primary barrier to their communication with patients,^11,29,30^ and there have been recent calls for oncologists to receive the training and knowledge to guide patients in integrative medicine.^31^ Moreover, studies find that oncologists consistently report a high level of interest in integrative approaches.^32,33^ Our findings are consistent with both of these lines of research, and the providers we interviewed reported high interest coupled with a perceived need to receive more information and education about the evidence base for major integrative approaches. The need for providers to feel comfortable initiating conversations about IO approaches is further highlighted by the consistent finding that patients are often reluctant to ask providers and rely instead on family or friends for information.^18,34^

Of note, we intentionally interviewed providers from across several disease categories. While a strength of this approach is that we can identify areas of wide consensus toward implementing an IO and Survivorship program, we did not attempt to identify differences between provider types. Previous studies have found wide variation in the extent to which providers recommend IO approaches to varying patient groups.^11^ While we are unable to determine whether providers we interviewed differ systematically, the notion that IO approaches should be targeted to specific cancer types was a strong theme and is consistent with this previous research.

By combining patient and provider perspectives, this study also revealed areas of potential mismatch that will be important to investigate further. Although integrating IO into patient downtime in the clinical environment was a relatively strong theme that emerged from the provider interviews, both active patients and survivors were more likely to indicate a preference for engaging with IO close to their homes. Understanding patient preferences in location for IO therapy will be critical, especially given the recent emphasis on embedding nonpharmaceutical approaches to depression, pain management, and QOL in “real-world” clinical settings and embedded into clinical care.^35,36^ Notably, although several survivors who were surveyed advocated for the importance of accessibility for those with physical disabilities, no providers mentioned this in the interviews. Identifying and evaluating ways to ensure that IO approaches are widely accessible is an important area of future research. Our study suggests that it may be under-appreciated by key stake-holders.

This study began as a comprehensive needs assessment to guide program development and implementation, and we are using these findings accordingly. For example, provider priorities around addressing cost as a key barrier, coupled with research on the successful implementation of shared medical visits for IO,^25^ shaped our early program implementation of shared medical appointments. These billable education appointments in which clinicians see multiple patients together in the same clinical setting are currently offered free of charge for oncology patients. These appointments have been held in a virtual setting, given the pandemic. Although it has thus far proved prohibitive to provide a safe, cancer-friendly environment and interventionists close to patients' homes in response to their reported preference, the virtual format of these group visits may also serve this preference.

Current research is underway to evaluate the implementation of this format, and other lines of research are emerging to suggest that virtual integrative approaches can be effectively implemented to address patient needs safely.^37^

A second way these findings have influenced program building is by focusing efforts to implement cost-effective acupuncture, motivated by patients' high interest levels in this integrative approach. Because acupuncture is not a billable service in our program and is often a financial burden to patients,^38^ we are developing a one-to-many model of group acupuncture that treats several patients in a group format to reduce out-of-pocket costs. Follow-up needs assessments, coupled with granular implementation research, will be vital toward understanding whether these approaches effectively address the needs and reduce the barriers identified in this initial study.

There are some limitations inherent to this study and our interpretation of the findings. As these data were collected in the initial stages of program implementation, we cannot make generalizable claims based on our findings. Our data were collected from patients and providers at a single NCI-designated cancer center, and previous research indicates that barriers to accessing IO modalities are steeper at community hospitals in comparison with Comprehensive Cancer Centers.^39^ In addition, we did not collect demographic data from the patient populations.

Previous research consistently finds that sociodemographic variables such as education, race, and sex/gender influence rates of and attitudes toward IO approaches.^18,40,41^ Moreover, we did not ask patients about their interest in using vitamins/minerals or food supplements, integrative approaches that have been shown in other studies to be highly popular.^18^ We also are unable to disambiguate patient preferences in virtual reality from their preferences in telehealth, as the two were combined into a single question. In future studies, we will also evaluate barriers from the patient and institutional perspective, which, in combination with the barriers identified in the provider interviews, can provide a more comprehensive approach toward implementation success.

Related to this future direction, we did not seek out and query providers who are antagonistic to IO interventions, and our positioning as program developers was known to the providers we interviewed. Both factors may have resulted in responses that were skewed toward supporting IO modalities with less critical feedback than is ideal for fully uncovering barriers.

Despite these limitations, this mixed-method study will shape the development and implementation of IO in several formative ways. Ultimately, these data highlight factors that can make IO easily accessible and acceptable to patients: providing services with an established evidence base that are targeted to patients' specific needs for symptom management and well-being; reducing burden by incorporating services into patient downtime; providing services accessible from patients' homes; and reducing or removing costs. Moreover, they point to factors that can make the implementation of IO services more successful: educating providers and staff about the evidence base for specific IO approaches; providing physicians, nurses, and APPs with decision trees, menu of services, and scripts that can be used with patients and their family; and understanding the local workflow to maximize staff and space.

## Supplementary Material

Supplemental data

## Abbreviations Used

APPs: advanced practice providers
CFIR: Consolidated Framework for Implementation Research
GU: genitourinary
GI: gastrointestinal
IO: integrative oncology
NCI: National Cancer Institute
QOL: quality of life
