# Supplementary material for: Examination of Provider and Patient Knowledge, Beliefs, and Preferences in Integrative Oncology at a National Cancer Institute-Designated Comprehensive Cancer Center
Source: Integr Med Rep. 2022 Jun 24;1(1):66–75. doi: 10.1089/imr.2021.0004 (PMC9372903; doi:10.1089/imr.2021.0004)
Supplement: Supplemental data [file Suppl_Data.pdf]

EMORY | WINSHIP CANCER INSTITUTE

A Cancer Center Designated by the National Cancer Institute

Integrative Oncology and Survivorship Survey

1. How familiar are you with complementary therapies (also known as Integrative Oncology, Holistic Medicine, or Complementary and Alternative Medicine)?

Not at all familiar — Extremely familiar

0	1	2	3	4	5	6	7	8	9	10

2. A summary of complementary therapies is listed below. If offered as part of your care, what services would you be interested in? (Please check all that apply).
   - ☐ Acupuncture
   - ☐ Acupressure
   - ☐ Massage Therapy
   - ☐ Yoga
   - ☐ Tai Chi
   - ☐ Nutrition
   - ☐ Spiritual Health Meditation/Mindfulness
   - ☐ Aromatherapy
   - ☐ Art Therapy
   - ☐ Music Therapy
   - ☐ Pet Therapy
   - ☐ Wellness and Exercise
   - ☐ Smoking Cessation
   - ☐ Educational Seminars
   - ☐ Child-life (children of survivors)
   - ☐ Counseling and Social Work
   - ☐ Oncology Support groups
   - ☐ None of the Above

3. What would be your preference?
   a. Individual therapy sessions
   b. Group therapy sessions

4. How often would be able to participate in complementary therapy?
   a. Once per week
   b. Twice per week
   c. Three times per week
   d. More than three times per week

Integrative Oncology and Survivorship Survey

5. How much time would you be able to dedicate to a single therapy session?

e. Less than 30 minutes
f. 30 minutes
g. 45 minutes
h. 60 minutes
i. More than 60 minutes

6. Where would you most likely attend complementary therapy?

a. Winship Cancer Institute
b. Closer to home
c. Online

7. If interested in complementary therapy, when would be the best time for you to attend sessions?

☐ On the day of your treatment / appointment at Winship Cancer Institute
☐ Weekdays during working hours (before 5pm)
☐ Weekdays in the evenings (after 5pm)
☐ Weekends
☐ No preference

8. Would you be interested in participating in complementary therapy using technology such as Virtual Reality or TeleHealth?

a. Yes
b. No
c. Unsure

9. Comments

Thank you for taking the time to complete the survey

Provider Questions

Friday, April 10, 2020
11:06 AM

Questions for Providers:

1. Introduction
2. We feel that provider input is critical in the implementation of this program and are calling to get your thoughts on integrative oncology?
3. What are the most important services that your patients can use?
   a. i.e. acupuncture, massage therapy, etc.
4. How do you think we can incorporate complementary medicine into your clinic?
5. What do you think are the most important aspects on integrative oncology?
6. What do you think are the education needs for our staff?
7. In terms of cost, some of these services may not be covered by insurance. How do you think we can assist our patients that lack the funds to get these services?
8. Are there are any gaps that you feel need to be addressed to implement this program?
9. Any other thoughts or recommendations?
